# A New Marker on Chicken Hematopoietic Cells is Defined by a Monoclonal Antibody Raised Against a V *ß* Chain of the Human TCR

**DOI:** 10.1155/1992/38159

**Published:** 1992

**Authors:** Anne-Sophie Lacoste-Eleaume, Christian Bleux, Catherine Corbel, Dominique Carriere, Philippe Poncelet, Jean Kanellopoulos, Colette Kanellopoulos-Langevin

**Affiliations:** 1 lnstitut Jacques Monod CNRS et Universitd Paris 7 2 Place Jussieu Tour 43 Paris Cedex 05 75251 France; 2 Institut d’Embryologie Cellulaire et Moléulaire CNRS et Collège de France 49 avenue de la Belle Gabrielle Nogent-sur-Marne 94736 France; 3 Centre de Recherches Sanofi Rue du Pr. J. Blayac Montpellier Cedex 34082 France; 4 Faculté de Médecine 27 boulevard Jean Moulin Marseille Cedex 05 13385 France; 5 Inserm U277 Institut Pasteur 25-28 rue du Dr. Roux Paris Cedex 15 75724 France

**Keywords:** Cross-reactive monoclonal antibody, T-cell receptor, chicken, hematopoietic cells, thrombocytes, erythrocytes

## Abstract

In this paper, we show that a mouse monoclonal antibody, 111-427, specific for the V *ß* 5.3 chain of the human T-cell receptor (TCR) for antigen, also reacts with chicken hematopoietic cells. Our data indicate that the majority of 111-427 positive cells among peripheral blood leucocytes (PBL) are thrombocytes. This antibody also recognizes two in vitro cell lines, III-C5, an IL-2-dependent T-cell-line and HD11, a macrophage cell line. In addition, erythrocytes and a minor subpopulation of thymus and spleen cells are also stained by the monoclonal antibody (mAb). No specific immunoprecipitation could be detected from ^125^I radiolabeled cell lysates. By Western blotting techniques, the 111- 427 mAb identifies a single band of apparent molecular weight 91 kD, unaffected by reduction, from III-C5 and HD11 cell lysates. This band is absent in negative cell control lysates. On thrombocytes, the apparent molecular weight of the band is shifted to 87 kD. These results indicate that the mAb does not recognize the chicken T-cell receptor for antigen, but a cell surface marker shared primarily between thrombocytes and erythrocytes. This new chicken cell marker is compared to other cell surface markers in avian or mammalian species that present some analogies in their tissue distribution.


*Developmental Immunology*, 1992, Vol. 2, pp. 273–284
Reprints available directly from the publisher
Photocopying permitted by license only

© 1992 Harwood Academic Publishers GmbH
Printed in the United Kingdom

### A New Marker on Chicken Hematopoietic Cells is Defined by a Monoclonal Antibody Raised Against a Vβ Chain of the Human TCR

ANNE-SOPHIE LACOSTE-ELEAUME,† CHRISTIAN BLEUX,† CATHERINE CORBEL,‡ DOMINIQUE CARRIERE,§ PHILIPPE PONCELET,# JEAN KANELLOPOULOS | | and COLETTE KANELLOPOULOS-LANGEVIN*†

*†Institut Jacques Monod, CNRS et Université Paris 7, 2 Place Jussieu, Tour 43, 75251 Paris Cedex 05, France*
*‡Institut d'Embryologie Cellulaire et Moléculaire, CNRS et Collège de France, 49 avenue de la Belle Gabrielle, 94736 Nogent-sur-Marne, France*
*§Centre de Recherches Sanofi, Rue du Pr. J. Blayac, 34082 Montpellier Cedex, France*
*#Faculté de Médecine, 27 boulevard Jean Moulin, 13385 Marseille Cedex 05, France*
*| | Inserm U277, Institut Pasteur, 25-28 rue du Dr. Roux, 75724 Paris Cedex 15, France*

In this paper, we show that a mouse monoclonal antibody, 111-427, specific for the Vβ 5.3 chain of the human T-cell receptor (TCR) for antigen, also reacts with chicken hematopoietic cells. Our data indicate that the majority of 111-427 positive cells among peripheral blood leucocytes (PBL) are thrombocytes. This antibody also recognizes two in vitro cell lines, III-C5, an IL-2-dependent T-cell line and HD11, a macrophage cell line. In addition, erythrocytes and a minor subpopulation of thymus and spleen cells are also stained by the monoclonal antibody (mAb). No specific immunoprecipitation could be detected from $^{125}$I radiolabeled cell lysates. By Western blotting techniques, the 111-427 mAb identifies a single band of apparent molecular weight 91 kD, unaffected by reduction, from III-C5 and HD11 cell lysates. This band is absent in negative cell control lysates. On thrombocytes, the apparent molecular weight of the band is shifted to 87 kD. These results indicate that the mAb does not recognize the chicken T-cell receptor for antigen, but a cell surface marker shared primarily between thrombocytes and erythrocytes. This new chicken cell marker is compared to other cell surface markers in avian or mammalian species that present some analogies in their tissue distribution.

KEYWORDS: Cross-reactive monoclonal antibody, T-cell receptor, chicken, hematopoietic cells, thrombocytes, erythrocytes.

### INTRODUCTION

The chicken immune system has been shown to possess two distinct lymphoid organs, the Bursa of Fabricius and the thymus, that have allowed the dichotomy between B and T lineages (Cooper et al., 1965). The chicken B-cell compartment is also unique in the generation of antibody diversity by gene conversion (Weill and Reynaud, 1987). The major histocompatibility complex (MHC) of the chicken, the B locus, is known to contain three genetic subregions identified as B-F, B-L, and B-G (Miller et al., 1988; Guillemot et al., 1989). The B-F and B-L subregions encode antigens that correspond to mammalian class I and class II antigens, respectively (Guillemot et al., 1988). The third region is tightly linked to B-F and B-L. At first, B-G antigens have been considered to be erythrocyte-specific (Longenecker and Mosmann, 1980; Salomonsen et al., 1987). But, by generating different hybridomas using erythrocyte-B-G molecules as immunogen, recent studies have demonstrated that B-G molecules and RNA are present in many other cell types: thrombocytes, peripheral B and T lymphocytes, bursal B cells and thymocytes and stromal cells in the bursa, thymus, and caecal tonsil of the intestine (Salomonsen et al., 1991). The exact role of these B-G molecules is unknown, but their extended tissue distribution suggests an important role in the immune system. So far, the B-G

*Corresponding author.

molecules are unique to birds because no analog has been found in other species. Nevertheless some approaches have shown that important structural subregions have been conserved between the human and avian species. For example, class II genes (B-L) of the chicken MHC have been investigated by Southern blot analysis using human cDNA probes (Anderson et al., 1987; Bourlet et al., 1988). Monoclonal antibodies to human and mouse MHC molecules have also been tested for binding to blood or spleen cells of different nonmammalian vertebrates (Kaufman et al., 1990). These monoclonal antibodies have allowed the identification of some MHC-like molecules in nonmammalian vertebrate species.

Monoclonal antibodies represent useful and powerful tools to study and characterize cell-surface antigens. The studies of T-cell compartment in chickens began with the production and characterization of mouse monoclonal antibodies that identify a variety of T-cell-surface molecules. These include homologs of the CD3 (Chen et al., 1986), CD4 (Chan et al., 1988), CD8 (Chan et al., 1988), and T-cell receptor molecules (Chen et al., 1988; Char et al., 1990; Cihak et al., 1988). Recently, another population of cytoplasmic CD3-positive cells that do not express the TCR was found in peripheral lymphoid organs of early chick embryos (Bucy et al., 1989). Monoclonal antibodies that react with new molecules in the chicken may be useful tools (1) to dissect cellular interactions during immune responses or inflammatory processes, (2) to investigate the functional activities of the different cell populations recognized, and (3) to follow such markers during embryonic development.

We have studied the reactivity of a mouse monoclonal antibody, namely, 111-427, on chicken cells. The 111-427 mAb has been initially produced and directed against the V$\beta$5.3 chain of the human T-cell lymphoma HPBALL. It has been found to recognize a subpopulation of blood leukocytes (2 to 10%) from healthy donors and allowed to immunoprecipitate the T-cell receptor from $^{125}$I surface-labeled HPBALL cells (Bleux et al., in preparation).

Our data demonstrate that this antibody has a different reactivity in chickens, because it stains several cell types of hematopoietic lineage, primarily thrombocytes and erythrocytes. Moreover, it recognizes a molecule of apparent molecular weight 87–91 kD (unaffected by reduction) in Western blot assays.

### RESULTS

### The Vast Majority of 11-427 Positive Leucocytes from Chicken Peripheral Blood Are Thrombocytes

Even though the 111-427 mAb has been shown to be specific for a subfamily of the receptor for antigen on human T lymphocytes, we found that its cell specificity was different in the chicken. We observed that the percentage of 111-427-positive cells in blood from the same chicken was dependent on the isolation procedure of white blood cells. Two different procedures for the preparation of PBL from whole chicken blood have been compared in terms of the yield of 111-427-positive cells. Enrichment of PBL over a Ficoll-Paque gradient yields a high percentage of thrombocytes (the nucleated cell equivalent to mammalian platelets) as previously described (Traill and Wick, 1983). Under these conditions, we have obtained a white cell population containing 65% of 111-427-positive white cells (Fig. 1A), whereas only 26% of cells express the CD3 marker of mature T lymphocytes (Fig. 1B). 97% of these cells bear the leucocyte common antigen recognized by HIS-C7 mAb with two main peaks of lower and brighter fluorescence (Fig. 1C).

In contrast, when buffy coat cells were stained after low-speed centrifugation, we have detected only 13% of 111-427-positive cells (Fig. 1D). Under these conditions, 63% of cells express the CD3 marker (Fig. 1E). All the cells were stained with HIS-C7 mAb, but with only one main peak of brightly stained cells (Fig. 1F). We have repeated this experiment over 20 times on different adult chicken strains (i.e., CC, CB, WB×M, White Leghorn, and Rhode Island chickens), and we have observed similar staining patterns, that is, the percentages of 111-427-positive cells is highly correlated with the enrichment in thrombocytic cells. These results indicate that the majority of 111-427-positive leucocytes in blood are thrombocytes. They have been confirmed in a second series of experiments in which Rhode Island thrombocyte-enriched leucocyte preparations were examined by two-color immunofluorescence with various combinations of the CT3 T-cell marker, a B-cell marker (detected by the M4 mAb), and the pan-leucocyte marker (Hist-C7). The 111-427-positive cells were found to be not associated with T-cell-marker expressing cells (Fig. 2B, 1% of double-stained cells is not significant on a 5,000-event acquisition) nor B-cell-marker positive cells (Fig. 2A) compared to the plots obtained with negative control mAb (Figs. 2D and 2E). However, the majority of the 111-427-positive cells belong to the leucocyte family (the weakly stained HIS-C7-positive cells, Fig. 2C).

These results show that the vast majority of 111-427-positive cells are neither T lymphocytes nor B lymphocytes, but belong to the leucocyte population, which includes thrombocytes.

### Morphological and Histological Analyses of 111-427-Positive Cells from Peripheral Blood

We have analyzed the histological cell types obtained after fluorescence-activated cell sorting of 111-427-positive cells from thrombocyte-enriched white-blood-cell preparations.

We observed nearly 100% of thrombocyte cell type, identified by May-Grünwald-Giemsa and morphological criteria as previously described (Lucas and Jamroz, 1961). We could also note the

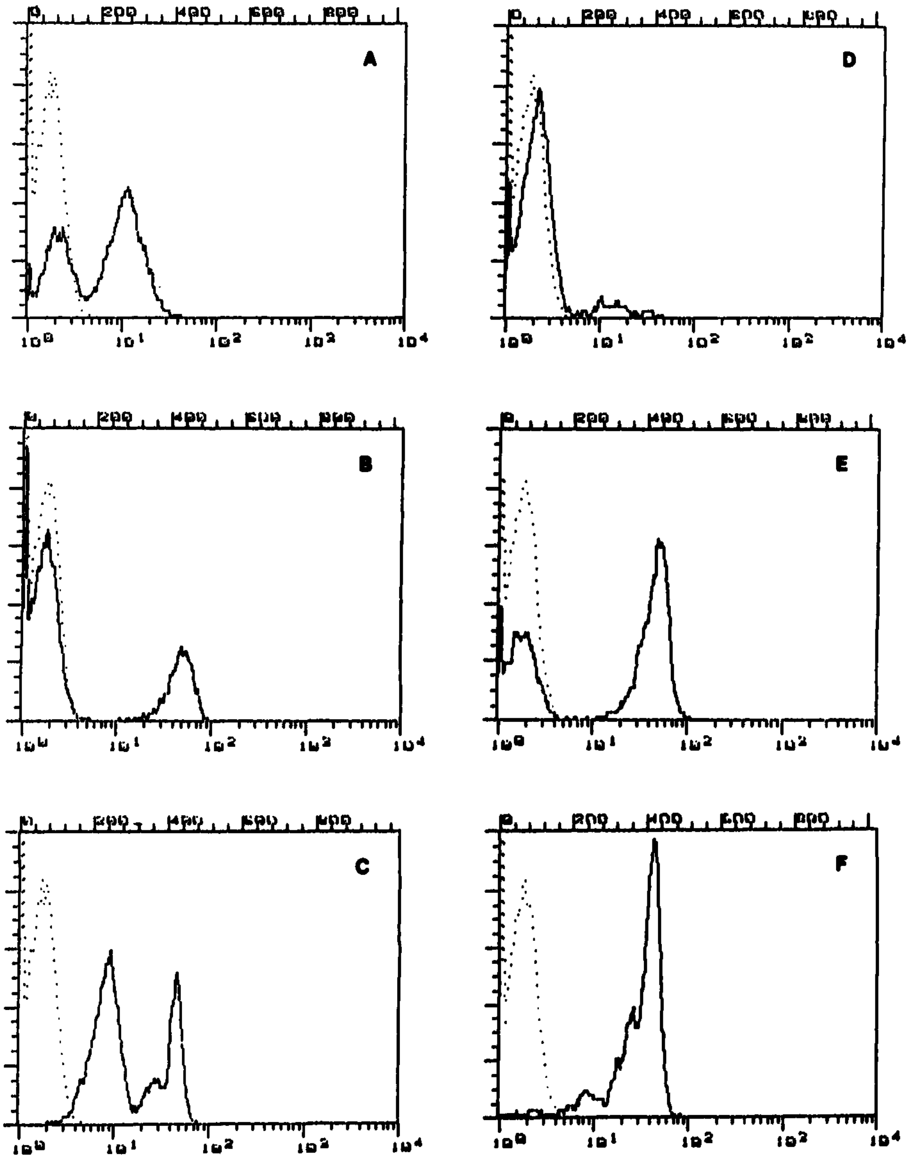

FIGURE 1. Influence of isolation procedures on the yield of 111-427-positive cells from the blood of adult Rhode Island chickens. Two isolation procedures have been compared: Ficoll-Paque gradient (A, B, C) and low-speed centrifuging (D, E, F). Primary antibodies are revealed by a FITC-conjugated goat anti-Ig. The relative intensity of green fluorescence is displayed on a log scale along the $x$ axis. The $y$ axis represents the number of cells. For each plot, dotted lines indicate staining of cells with a control IgG1 mAb. Solid lines indicate the staining intensity with 111-427 (A, D), CT3 (B, E), and HIS-C7 (C, F).

presence of a few contaminating erythrocytes in the 111-427-positive population (Fig. 3A). The thrombocytes had densely clumped chromatin in a slightly oval nucleus. The same histological analysis on negative sorted cells revealed the presence of a majority of lymphocytes and a few monocytes (Fig. 3B).

Thus, the staining of blood thrombocytes by the 111-427 mAb has been confirmed by histological and morphological criteria.

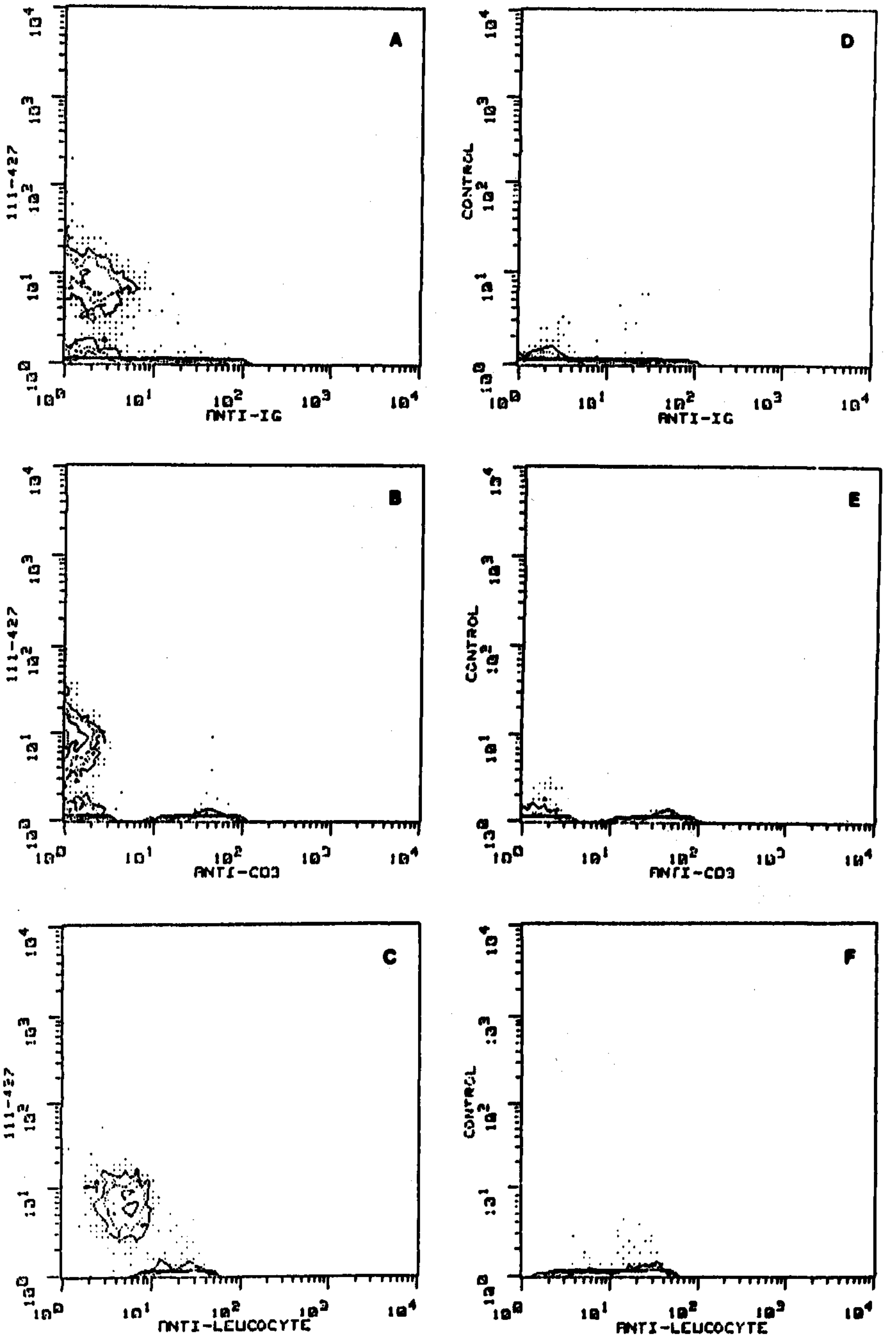

FIGURE 2. Two-color immunofluorescence analysis of peripheral white blood cells from a Rhode Island chicken. Blood cells prepared over a Ficoll-Paque gradient were first stained with unconjugated M4 (A, D) or CT3 (B, E) or HIS-C7 (C, F) mAb followed by FITC- conjugated goat anti mouse Ig. The relative intensity of green fluorescence is displayed on a log scale along the $x$ axis. Then, in the presence of saturating amounts of mouse Ig (1 mg/ml), control (D, E, F) or 111-427 (A, B, C) biotinylated mAb were added, followed by PE-conjugated streptavidin. The relative intensity of red fluorescence is displayed on a log scale along the $y$ axis.

### Tissue Distribution of Cells Reactive with the 111-427 mAb

### Red blood cells

The presence of erythrocytes among 111-427-positive sorted cells led us to check whether it was a specific labeling. Therefore, we have enriched erythrocytes from blood and stained them with the mAb. Figure 4 shows that the great majority of chicken red cells are stained by this antibody.

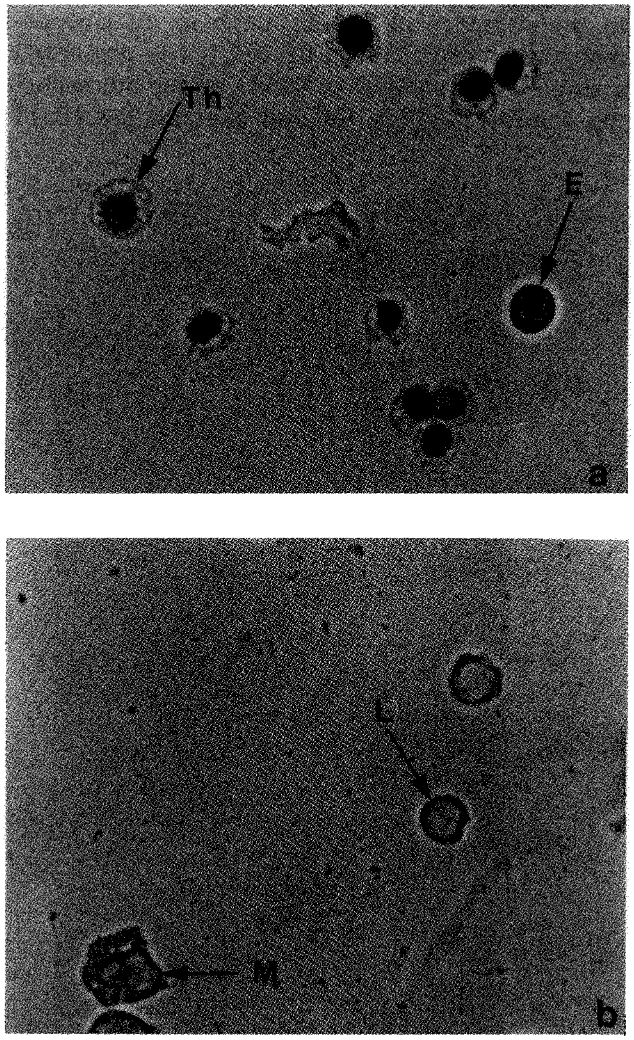

FIGURE 3. Thrombocyte and erythrocyte staining by 111-427 mAb. Cytocentrifuge cell preparations of sorted 111-427-positive (a) and -negative (b) cells from PBL obtained by Ficoll preparations. May-Grünwald-Giemsa staining. Magnification: x 970. (a) 111-427-positive cells; Th: thrombocyte; E: erythrocyte. (b) 111-427-negative cells; L: lymphocyte; M: monocyte.

### Lymphoid organs

Single cell suspensions from lymphoid organs taken from White Leghorn or Rhode Island chickens were stained with the 111-427 mAb and a panel of mAb directed against chicken lymphoid cell markers. Cells were examined by flow cytometry. As shown in Table 1, expected percentages of positive cells were obtained with antibodies against chicken CD3 (Chen et al., 1986), CD4, and CD8 (Chan et al., 1988) markers. Moreover, a low but significant and reproducible number of white cells from lymphocyte-enriched blood spleen, and thymus was labeled by the 111-427 mAb. No staining was observed in the Bursa of Fabricius, no matter the age of the animal (data not shown). We have made at least 10 different determinations, and no significant variation in the percentage of 111-427-positive cells was observed between chickens taken from age 4 days up to 8 weeks.

### In vitro cell lines

The specificity of the 111-427 mAb was also assayed on various in vitro cell lines by indirect immunofluorescence. A typical experiment is presented in Table 2. The 111-427 mAb was found to stain 100% of III-C5 T-cell and 100% of a macrophage cell line HD11. It did not stain a B

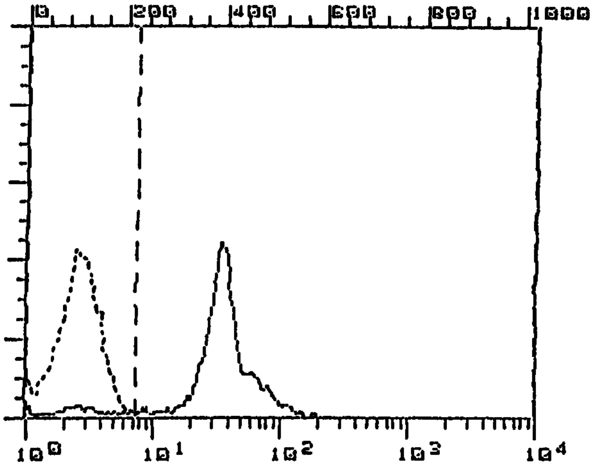

FIGURE 4. Flow cytometry analysis of 111-427 mAb expression on adult peripheral blood erythrocytes. Green fluorescence is displayed on a log scale. The $x$ axis represents the relative fluorescence intensity versus the number of cells along the $y$ axis. Solid lines indicate staining with 111-427 mAb and dotted lines staining with negative control mAb.

lymphoblast, a myeloblast, a monoblast, an erythroblast, and a T lymphoblast tested in the same experiment.

### Biochemical Characterization of the Molecule Recognized by the 111-427 mAb

In order to characterize the molecule(s) recognized by the 111-427 mAb, we first tried to immunoprecipitate $^{125}I$ surface-labeled purified blood leucocytes or III-C5 cells. One-dimensional gel electrophoresis did not reveal any specific band from the 111-427 immunoprecipitates, whereas other cell-specific antibodies (included as positive controls in every experiment) gave expected results. We also performed two-dimensional nonreducing/reducing SDS-PAGE analyses of our immunoprecipitates. Again, no specific spot was detected from 111-427 immunoprecipitates in experiments in which anti-CD3 and anti-CD4 antibodies yielded unequivocally positive results (data not shown).

We then performed a Western blot technique that allows the detection of antigens by lower-affinity antibodies. As shown in Fig. 5, various concentrations of 111-427 mAb revealed a single band of 91 kD from III-C5 cell lysate under reducing conditions (lanes A, B, and C). The intensity and the width of the band were dose-dependent, with the appearance of some background staining at the largest dose tested (lane A). Similar concentrations of an isotype-matched control antibody (the anti-CD3 antibody does not work in Western blotting assays) gave no signal (lanes D, E, and F). A similar band was observed from III-C5 cell lysate run under nonreducing conditions (Fig. 6). In Fig. 6 are presented the results of a typical Western blot assay in which we have tested three in vitro cell lines in parallel; two are stained by the 111-427 mAb (III-C5 and HD11) and the third one is 111-427-negative (MSB1) (cf. Table 2). Each cell lysate was assayed in the presence of 111-427 mAb or an isotype-matched control. The III-C5 cell lysate presented a single specific band of 91 kD under nonreducing conditions (Figs. 6A and 6B) and the HD11

TABLE 1
Immunofluorescence Staining of Different Lymphoid Tissues

| Organs | Monoclonal antibodies | | | |
|---|---|---|---|---|
| | 111-427 | CT3 | CT4 | CT8 |
| Spleen | 12±7[a] | 55±12 | 20±7 | 37±6 |
| Blood[b] | 8±5 | 64±10 | 37±14 | 15±5 |
| Thymus | 6±3 | 35±7 | 71±4 | 65±3 |

[a]The results are expressed as percentages of positive cells determined by indirect immunofluorescence by flow cytometry, means of 10 experiments±standard error of the mean from chickens aged 4 days to 8 weeks.

[b]Blood cells are prepared by low-speed centrifuging.

TABLE 2
Reactivity of 111-427 mAb with Various In Vitro Cell Lines[a]

| Cell line | Cell type | Positive cells |
|---|---|---|
| RPL12 | B lymphoblast | 0 |
| MSB1 | T lymphoblast | 0 |
| III-C5 | Activated T cell | 100 |
| HD3 | Erythroblast | 0 |
| HD11 | Macrophage | 100 |
| E26 | Myeloblast | 0 |
| BM2C3 | Monoblast | 0 |

[a]The results are expressed as a percentage of positive cells determined by indirect immunofluorescence and measured by flow cytometry.

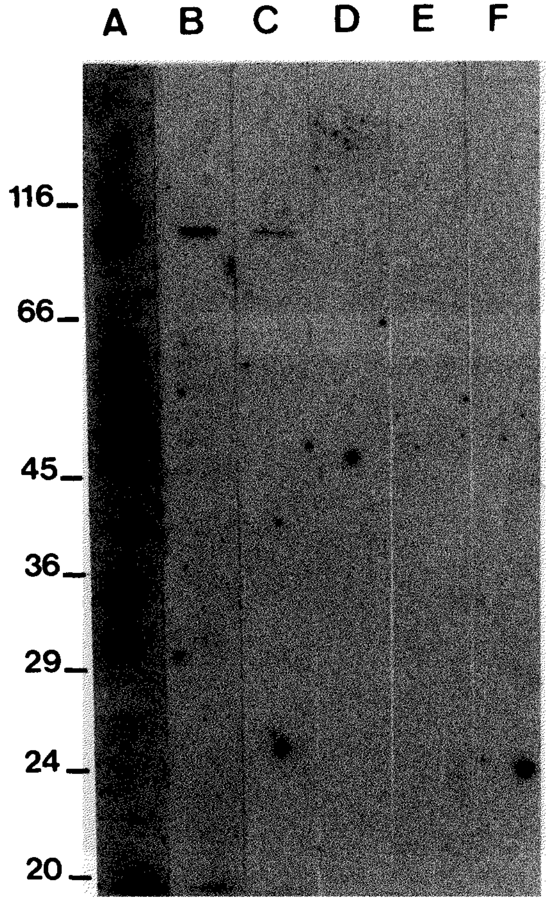

FIGURE 5. Western blots on III-C5 cell lysates, under reducing conditions, with various concentrations of 111-427 mAb (i.e., A: 0.07 mg/ml; B: 0.02 mg/ml; and C: 0.008 mg/ml) compared with different dilutions of CT3 supernatant (i.e., D: 1/10; E: 1/40; and F: 1/100).

cell lysate gave an identical result (Figs. 6E and 6F). In contrast, no band was present in the MSB1 tracks (Figs. 6C and 6D). In order to see whether our antibody recognized the same molecule on the surface of blood thrombocytes, we assayed the cell lysates from thrombocyte-enriched preparations from four different chicken strains, that is CC, CB, WB×M, and Rhode Island. The two former strains are homozygous at their MHC locus, B4 and B12, respectively. As shown in Fig. 7, under nonreducing conditions, all four thrombocyte lysates produced a single specific band of 87 kD (lanes c, e, g, and i). In the same experiment, the usual 91-kD band was obtained from the III-C5 lysate (lane a). Similar results were obtained under reducing conditions (data not shown). The slight size difference found in the thrombocyte lysates compared to the two cell lines might be due to different glycosylations. In contrast, no size differences were observed among thrombocytes from various MHC-differing chicken strains.

In summary, using a Western blot assay, we have shown that the molecule recognized by the 111-427 mAb on chicken hematopoietic cells yields a single band of apparent molecular weight 87 to 91 kD under reducing or nonreducing conditions.

### DISCUSSION

On human leucocytes, the 111-427 mAb reactivity is restricted to a subpopulation of T lymphocytes bearing the Vβ5.3 chain of the human TCR (Bleux et al., in preparation). In contrast, the present analysis of the reactivity of this mAb in chickens shows a very distinct pattern, with a molecule of 87–91 kD being recognized on several different cell types. This activity is removed after absorption on 111-427-positive HPBALL human T-lym-

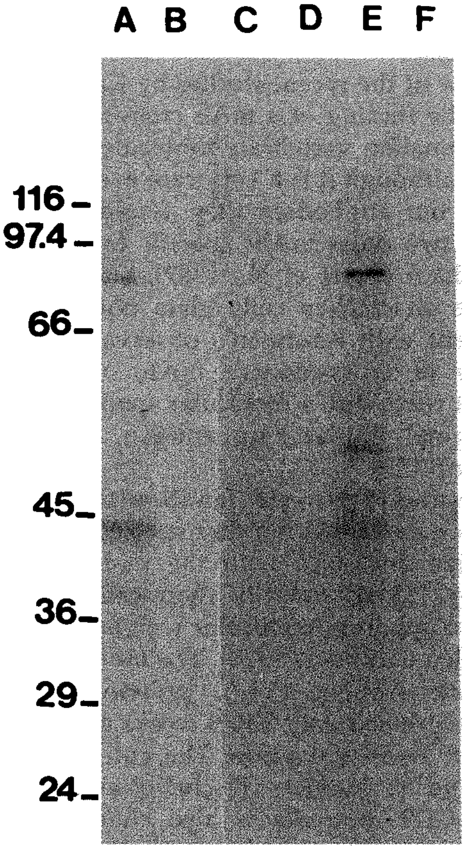

FIGURE 6. Western blots on III-C5 (A, B), MSB1 (C, D) and HD11 (E, F) cell lysates, with 0.03 mg/ml 111-427 mAb (A, C, E) compared with control IgG1 mAb (B, D, F). This experiment was performed under nonreducing conditions.

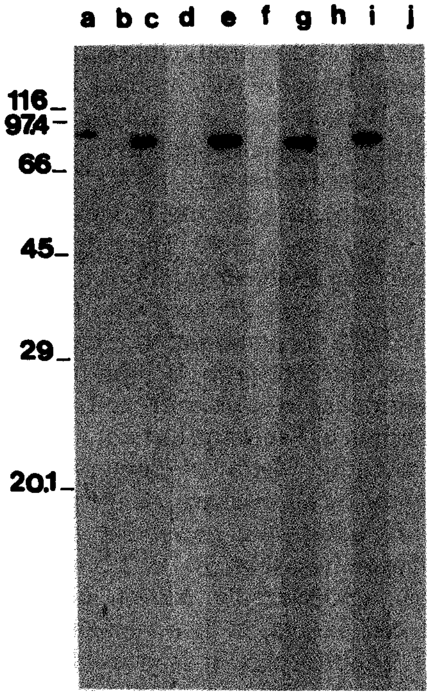

FIGURE 7. Western blots on thrombocyte lysates from different strains under reducing conditions: CC (lanes c, d), CB (lanes e, f), WB/M (lanes g,h), Rhode Island (lanes i, j) compared to III-C5 cell lysate (lanes a, b) either with 0.03 mg/ml 111-427 mAb (lanes a, c, e, g, i) or control IgG1 mAb (lanes b, d, f, h, j).

phoma cells (data not shown). The major cell type recognized among chicken white blood cells is thrombocytes. Three separate lines of evidence support this conclusion: (1) a preparation procedure described to enrich leucocyte populations in thrombocytes reproducibly leads to a parallel increase in the percentage of 111-427-positive cells measured by direct or indirect immunofluorescence (this is in agreement with the work of Traill and Wick (1983) who showed that the yield in lymphocytes versus thrombocytes from whole blood depends on the procedure used for the preparation of peripheral white blood cells); (2) by two-color immunofluorescence staining, the vast majority of 111-427-positive cells double-stains with a pan leucocyte marker, but not with mature T- or B-lymphocyte markers; (3) by histological and morphological criteria, the great majority of 111-427-positive fluorescence-activated sorted cells are thrombocytes and not lymphocytes or monocytes. In addition, we have observed, by flow cytometry and microscopy, that chicken erythrocytes are also stained by this antibody. Immunofluorescence staining on lymphoid organs from outbred chickens taken from 4 days to 8 weeks of age shows that a low but significant and reproducible number of cells from spleen, lymphocyte-enriched blood leucocytes, and thymus was labeled. In these experiments, the percentages of positive cells observed with control antibodies (i.e., CT3, CT4, and CT8) were in agreement with the literature (Chen et al., 1986; Chan et al., 1988). The gating set up on the fluorescence analyzer rules out the possibility that the positive results with the antibody are due to red-cell contamination. Some contamination with thrombocytes is more likely. However, preliminary results with cell preparations from the Bursa of Fabricius did not show any significant binding, with an otherwise normal expression of B-cell markers. At the present time, we cannot exclude the possibility that a minor subpopulation (less than 5%) of T lymphocytes or monocytes, undetectable within the sensitivity limits of our assays, is also recognized. In the case of T lymphocytes, it is possible that these cells express the epitope after activation only, which would go along with the recognition of the III-C5-activated T-cell line (Corbel and Thomas, 1990). In contrast, the MSB1 cell line we have tested is MHC-class II-negative and IL-2-receptor negative. Preliminary results on 48-hr Con A-activated peripheral blood leucocytes or 5-day mixed lymphocyte reaction blasts (data not shown) were negative, but it may be necessary to check long-term activated T cells from blood or spleen before any definitive conclusion can be reached.

The biochemical characterization of the molecule recognized by the 111-427 mAb could be achieved only by using a sensitive Western blot assay, as all attempts to immunoprecipitate the molecule were unsuccessful, probably because of the low affinity of the antibody for its ligand, once in detergent solutions. Only fluorescence-positive cell lines or tissues produced a single band of apparent molecular weight 91 kD (unaffected by reduction), for two in vitro cell lines. Thrombocytes presented a slightly lower molecular weight of 87 kD, whatever the MHC haplotype of the chicken strain. Thus, although the tissue distribution could indicate a similarity with a B-G molecule (Salomonsen et al., 1991), the biochemical data do not support this hypothesis.

In view of the recent studies by Kaufman et al. (1990), the finding of a mAb cross-reacting with nonmammalian vertebrate molecules is not surprising, although it is a very rare event (less than 10% of the antibodies they tested). We have assayed two other mAbs specific for the human V$\beta$5.3 chain (Borst et al., 1987), and they were negative on chicken cells (data not shown). In agreement with Kaufman's observations on the majority of cross-reactive mAb, our antibody does not react with a molecular species similar to the original immunizing antigen, that, is the human $\alpha\beta$ TCR.

Interestingly, several families of molecules have been described in other species, which present some analogies with the molecule recognized by 111-427 mAb in their cell-type distribution or apparent molecular weights (cf. review by Albelda and Buck, 1990). The molecules of the Very Late Antigen (VLA) protein family are highly expressed only 2 to 3 weeks after the triggering of T-cell activation. The antigen VLA2 is also found on platelets (Pischel et al., 1987). The human CD9 molecule is also shared between platelets and a variety of hematopoietic and nonhematopoietic tissues. Antibodies against CD9 have been used to trigger platelet activation and granule secretion (Carroll et al., 1990). Some other cell adhesion molecules with comparable properties are also members of the immunoglobulin gene superfamily, such as LFA-3, the ligand of CD2, which is present on both hematopoietic (including red blood cells) and nonhematopoietic cell lineages. In the same family, Newman et al. (1990) have identified a 130-kD glycoprotein on human cells, PECAM-1, or endoCAM on bovine cells, which are found on platelets, blood monocytes, neutrophils, mitogen-induced lymphoblasts, and endothelial cells. Nevertheless, no striking similarity with any of these molecules has emerged, so that we cannot at present relate this new chicken marker to any known surface molecule in the mammalian species.

We are extending our analysis of the expression of the 111-427 epitope to nonhematopoietic tissues and also during the embryonic development. Attempts to activate thrombocytes or monocytes with the antibody are in progress and our main goal is to clone this molecule.

Although such studies are required to elucidate the possible functional role of the molecule, as well as to determine its sequence and primary structure, our present data indicate it could be a useful new cell marker in the chicken.

### MATERIALS AND METHODS

### Animals

Adult and newborn, outbred White Leghorn and Rhode Island chickens were obtained from a local commercial source. Chickens from inbred lines CC, CB, and a F1 cross between WB and M, respectively, the B4, B12, and the B15×B21 MHC haplotypes (Briles and Briles, 1982) were raised at the Institut d'Embryologie Cellulaire et Moléculaire, Nogent-sur-Marne (France) from fertilized eggs provided by Dr. Hlozanek (Institute of Molecular Genetics, Prague).

### Cell Lines

We have used an activated helper T-cell line III-C5 (Corbel and Thomas, 1990) and six transformed cell lines. MSB1, a Marek's Disease Virus (MDV)-transformed T-lymphoblast cell line (Akiyama and Kato, 1974), RPL12, a Rous-associated Avian Virus (RAV)-induced lymphoblastoid B-cell line (Okazaki et al., 1980), HD11, a MC29-transformed macrophage cell line (Beug et al., 1979), HD3, an Avian Erythroblastosis Virus (AEV)-transformed erythroblast cell line (Beug et al., 1979), E26, an E26 myeloblastosis virus-transformed myeloblast cell line (Beug et al., 1979), and BM2C3, an Avian Myeloblastosis Virus (AMV)-transformed monoblast cell line (Moscovici et al., 1982; Moscovici, 1985). They were maintained in Dulbecco's modified Eagle's medium with 10% heat-inactivated fetal calf serum (FCS-C', Eurobio laboratories, Paris), 1% chicken serum, 2-mM L-glutamine, and 50-mg/ml penicillin/streptomycin at 40 °C in a 5% $CO_2$ atmosphere. III-C5 cells are IL-2-dependent and were cultured in Iscove's modified Dulbecco's Medium with 10% of FCS-C' and 10% IL-2-containing medium as described previously (Corbel and Thomas, 1990).

### Cell Preparations

Chickens were bled from the wing vein on Heparin-containing buffer (Calciparine Choay, Becton Dickinson, USA, 10 U/ml). Peripheral blood leucocytes were obtained either by low-speed centrifugation or Ficoll-Paque gradient.

Low-speed centrifugation (lymphocyte enrichment): Centrifugation of whole blood at room temperature at 62 g for 20 min and collection of buffy coat cells.

Ficoll-Paque gradient (thrombocyte enrichment): Centrifugation of whole blood over a Ficoll-Paque density gradient (Pharmacia Fine Chemicals, Uppsala, Sweden, 1.077 g/l) at 250 g for 10 min at room temperature and collection of the interphase cells. The procedure was repeated twice.

The cells were washed three times in Medium 199 (Eurobio Laboratories, Paris) supplemented with 5% of FCS-C .

Erythrocytes were obtained by centrifugation at 800 g from whole blood followed by three washes in Medium 199.

Thymus, spleen, and bursa cell suspensions were prepared by gently teasing the organs on a sieve in Medium 199 plus 5% FCS-C'. Viable splenic cells were isolated by centrifugation over Ficoll-Paque. The final cell viability determined by trypan blue exclusion was superior to 90% for all tissue samples.

### Immunofluorescence Staining

For indirect immunofluorescence, the cells (pellet of $0.5\times10^6$ cells) were incubated with various monoclonal antibodies (2.5 $\mu$g for purified antibody or 25 $\mu$l for culture supernatant) for 30 min at room temperature for cultured cells or at 4 °C for other cells. Then the cells were washed twice with the following buffer: Hank's Balanced Salt Solution (HBSS) supplemented with 2.5% of FCS-C' 5% of sodium pyruvate, and 0.2% $NaN_3$. The cells were then incubated with fluorescein (FITC)-conjugated goat antimouse Ig antibody (Euromedex, France) or phycoerythrin (PE)-conjugated goat antimouse Ig antibody (Southern Biotechnology Associates, Alabama) for 30 min.

For two-color immunofluorescence, the cells were incubated with unconjugated mAb followed by staining with FITC-conjugated goat antimouse Ig antibody for 30 min. Then normal mouse immunoglobulins were added to block-free sites of FITC-anti-Ig (1 mg/ml) followed by staining with the second biotinylated mAb and PE-conjugated streptavidin (Southern Biotechnology Associates).

Fluorescence stainings were measured and positive cells enumerated by flow cytometry on a FACScan (Becton Dickinson) in Prof. M. Kazatchkine's laboratory (Hospital Broussais, Paris). For each plot, analyses were made on 5,000 gated events. The gating is based on forward and right-angle side-scatter parameters that take into account cell size, viability, and granulosity. For all the experiments, the cells were also examined with a microscope equipped with epifluorescence optics.

Sorted Ficoll-separated peripheral blood leucocytes were done on FACS 440 with P. Vaigot (Institut d'Embryologie Cellulaire et Moléculaire, Nogent-sur-Marne).

### Antibodies

Monoclonal antibodies used in this study were as follows: (1) 111-427, an $IgG_1\kappa$ subclass mAb directed against the V$\beta$5.3 chain of the human TCR for antigen (Bleux et al., in preparation). (2) CT3 (antichicken CD3, Chen et al., 1986), CT4 (antichicken CD4, Chan et al., 1988), CT8 (antichicken CD8, Chan et al., 1988), and M4 (antichicken $\mu$ chain, Chen et al., 1982) were kindly provided by Dr. M. Cooper (Birmingham, Alabama). (3) HIS-C7, a common leucocyte marker (Jeurissen et al., 1988) was given by Dr. S. Jeurissen (Lelystad, The Netherlands). (4) An isotype-matched $IgG_1\kappa$ mAb directed against the $\beta$-galactosidase from Escherichia coli and nonreactive with chicken cells was used as control. It was the kind gift of Prof. C. Le Guern (Institut Pasteur, Paris).

### Biochemistry Assays

### Preparation of cell lysates for Western Blot analysis

Method used for cell lines (i.e., III-C5, HD11, and MSB1): $10^8$ cells were solubilized in sample buffer, 2% SDS, 60 mM Tris pH 6.8, 10% glycerol, 0.01% bromophenol blue, containing protease inhibitors and mixed vigorously. The samples were then boiled for 5 min. The chromosomal DNA was sheared by passing the sample repeatedly through a 20-gauge needle and then through a 26-gauge needle. The samples were centrifuged at 10,000 g from 10 min; the supernatant was recovered. Then the samples were applied to SDS gels or frozen at –80 °C until used.

Method used for thrombocytes: $2\times10^8$ thrombocytes were solubilized in 500-$\mu$l 1% NP-40 lysis buffer containing protease inhibitors on ice for 30 min and ultracentrifuged (100,000 g for 1 hr at 4 °C). The supernatant was boiled with 500-$\mu$l SDS sample buffer with or without 5% $\beta$-mercaptoethanol. Then the samples were applied to acrylamide gels in SDS or frozen at –80 °C if not used immediately.

### Gel Electrophoresis and Western Blot Analysis

The lysates were applied to a 10% acrylamide gel in SDS-PAGE under nonreducing or reducing conditions. Molecular weight markers (MWM) used for SDS gel electrophoresis are $\beta$-galactosidase 116 kD, phosphorylase 97.4 kD, bovine albumin 66 kD, egg albumin 45 kD, glyceraldehyde 3-phosphate deshydrogenase 36 kD, carbonic anhydrase 29 kD, trypsinogen 24 kD, and soybean trypsin inhibitor 20.1 kD (MW-SDS 200 and MW-SDS 70L kits, Sigma, France).

Western Blot assays were performed using the Enhanced Chemiluminescence (ECL) Western blotting detection system (RPN 2106, Amersham, France). After electrophoresis, protein were transferred to 0.45-$\mu$m nitrocellulose membrane (RPN 303E, Amersham, France) in transfer buffer (25 mM Tris, 190 mM glycine, 20% methanol v/v, pH 8.6) at 0.4 A for 4 hr. After blotting, the nitrocellulose was cut into strips and incubated for 1 hr at room temperature in blocking buffer (10% w/v nonfat dry milk, 0.2% Tween 20 in PBS). The procedure was repeated twice. Each antibody was then incubated (30 μg/ml) with the membrane strips overnight at 4 °C. The strips were washed five times with PBS+0.5% Tween 20 before incubation with a goat antimouse IgG (H+L) peroxidase conjugate (Promega, USA) for 2 hr at room temperature, followed by five more washes. The strips were incubated in detection reagents. The goat antimouse IgG peroxidase conjugate catalyses the oxidation of luminol, which, in the presence of a chemical enhancer, produces a sustained light emission: ECL. The strips are then exposed for autoradiography in a metal cassette for 1 to 10 min according to the intensity of the reaction.

### ACKNOWLEDGMENTS

The authors wish to thank Drs. M.D. Cooper, C.L. Chen, S. Jeurissen, and C. Le Guern for their generous supply of reagents, Prof. M. Kazatchkine for the free access to his flow cytometry facility in Hospital Broussais, Mr. P. Vaigot (Nogent-sur-Marne) for the cell-sorting experiments, and Mr. B. Henri (Nogent-sur-Marne) and Mr. R. Schwartzmann (Institut Jacques Monod, Paris) for the photographs. The helpful discussions with Drs. M.D. Cooper and C.L. Chen are also gratefully acknowledged.

This work was supported in part by the Institut National de la Santé et de la Recherche Médicale (INSERM), and the Fondation pour la Recherche Médicale. A.S. Lacoste-Eleaume has a fellowship from the French Ministry of Research and Technology.

*(Received December 9, 1991)*

*(Accepted January 28, 1992)*

### REFERENCES

Akiyama Y., and Kato S. (1974). Two cell lines from lymphomas of Marek's disease. Biken J. **17:** 105–116.

Albelda S.M., and Buck C.A. (1990). Integrins and other cell adhesion molecules. Faseb J. **4:** 2868–2880.

Anderson L., Lunberg C., Rask L., Gissel-Nielsen B., and Simonsen M. (1987). Analysis of class II genes of the chicken MHC B by use of humán DNA probes. Immunogenetics **26:** 79–84.

Beug H., Von Kirchbach A., Döderlein G., Conscience J.F., and Graf T. (1979). Chicken hematopoietic cells transformed by seven strains of defective avian leukemia viruses display three distinct phenotypes of differentiation. Cell **18:** 375–390.

Borst J., De Vries E., Spits H., De Vries J., Boylston A.W., and Matthews E.A. (1987). Complexity of T cell receptor recognition sites for defined alloantigens. J. Immunol. **139:** 1952–1959.

Bourlet Y., Béhar G., Guillemot F., Fréchin N., Billault A., Chaussé A.M., Zoorob R., and Auffray C. (1988). Isolation of chicken major histocompatibility complex class II (B-L) β chain genes: Comparison with mammalian β chains and expression in lymphoid organs. EMBO J. **7:** 1031–1039.

Briles W.E., and Briles R.W. (1982). Identification of haplotypes of the chicken major histocompatibility complex (B). Immunogenetics **15:** 449–459.

Bucy R.P., Coltey M., Chen C.L., Char D., Le Douarin N.M., and Cooper M.D. (1989). Cytoplasmic CD3+ surface CD8+ lymphocytes develop as a thymus-independent lineage in chick-quail chimeras. Eur. J. Immunol. **19:** 1449–1455.

Carroll R., Worthington R., and Boucheix C. (1990). Stimulus-response coupling in human platelets activated by monoclonal antibodies to the CD9 antigen, a 24 kDa surface-membrane glycoprotein. Biochem. J. **266:** 527–535.

Chan M.M., Chen C.L., Lanier Ager L., and Cooper M.D. (1988). Identification of the avian homologues of mammalian CD4 and CD8 antigens. J. Immunol. **140:** 2133–2138.

Char D., Sanchez P., Chen C.L., Bucy R.P., and Cooper M.D. (1990). A third sublineage of avian T cells can be identified with a T-cell receptor-3 specific antibody. J. Immunol. **145:** 3547–3555.

Chen C.L., Cihak J., Lösch U., and Cooper M.D. (1988). Differential expression of two T cell receptors, TCR1 and TCR2, on chicken lymphocytes. Eur. J. Immunol. **18:** 539–543.

Chen C.L., Lanier Ager L., Gartland G.L., and Cooper M.D. (1986). Identification of a T3/T cell receptor complex in chickens. J. Exp. Med. **164:** 375–380.

Chen C.L., Lehmeyer J.E., and Cooper M.D. (1982). Evidence for an IgD homologue on chicken lymphocytes. J. Immunol. **129:** 2580–2585.

Cihak J., Ziegler-Heitbrock H.W.L., Trainer H., Schranner I., Merkenschlager M., and Lösch U. (1988). Characterization and functional properties of a novel monoclonal antibody which identifies a T-cell receptor in chicken. Eur. J. Immunol. **18:** 533–537.

Cooper M.D., Peterson R.D.A., and Good R.A. (1965). Delineation of the thymic and bursal lymphoid systems in the chicken. Nature **205:** 143–146.

Corbel C., and Thomas J.L. (1990). Establishment of an IL-2 dependent, antigen non-specific chicken T-cell line. Dev. and Comp. Immunol. **14:** 439–446.

Guillemot F., Billault A., Pourquié O., Béhar G., Chaussé A.M., Zoorob R., Kreibich G., and Auffray C. (1988). A molecular map of the chicken major histocompatibility complex: The class II β genes are closely linked to the class I genes and the nucleolar organizer. EMBO J **7:** 2775–2785.

Guillemot F., Kaufman J., Skjoedt K., and Auffray C. (1989). The major histocompatibility complex in the chicken. Trends Genet. **5:** 300–304.

Jeurissen S., Janse E.M., Ekino S., Nieuwenhuis P., Koch G., and De Boer G.F. (1988). Monoclonal antibody as probes for defining cellular subsets in the bone marrow, thymus, Bursa of Fabricius and spleen of the chicken. Vet. Immunol. Immunopathol. **19:** 225–238.

Kaufman J., Ferrone S., Flajnik M., Kilb M., Völk H., and Parisot R. (1990). MHC-like molecules in some non-mammalian vertebrates can be detected by some cross-reactive monoclonal antibodies. J. Immunol. **144:** 2273–2280.

Longenecker B.M., and Mosmann T.R. (1980). Restricted expression of an MHC alloantigen in cells of the erythroid series: A specific marker for erythroid differentiation. J. Supramolec. Struc. **13:** 395–400.

Lucas A., and Jamroz C. (1961). Atlas of avian hematology. (Washington, D.C.: U.S. Department of Agriculture).

Miller M., Goto R., and Briles W.E. (1988). Biochemical confirmation of recombination within the B-G subregion of the chicken major histocompatibility complex. Immunogenetics **27:** 127–132.

Moscovici C. (1985). Target cells for avian leukemia viruses revisited. *In:* Retroviruses and human pathology, Gallo R.C., Stehelin D., and Vamier, O.E. Eds. (New York: The Humana Press), pp. 177–191.

Moscovici C., Zeller N., and Moscovici M.G. (1982). Continuous lines of AMV-transformed non-producer cells: Growth and ontogenic potential in the chick embryo. *In:* Expression of differentiated functions in cancer cells, Revoltella R.F. et al., Eds. (New York: Raven Press), pp. 435–449.

Newman P., Berndt M., Gorsky J., White G., Paddock L., and Muller W. (1990). PECAM-1 (CD31) cloning and relation to adhesion molecules of the immunoglobulin gene superfamily. Science **247:** 1219–1222.

Okazaki W., Witter R.L., Romero C., Nazerian K., Sharma J.M., Fadly A., and Ewert D. (1980). Induction of lymphoid leukosis transplantable tumors and the establishment of lymphoblastoid cell lines. Avian Pathol. **9:** 311–329.

Pischel K., Hemler M., Huang C., Bluestein H., and Woods V. (1987). Use of monoclonal antibody 12F1 to characterize the differentiation antigen VLA-2. J. Immunol. **138:** 226–233.

Salomonsen J., Skjodt K., Crone M., and Simonsen M. (1987). The chicken erythrocyte-specific MHC antigen. Characterization and purification of the B-G antigen by monoclonal antibodies. Immunogenetics **25:** 373–382.

Salomonsen J., Dunon D., Skjodt K., Thorpe D., Vainio O., and Kaufman J. (1991). Chicken major histocompatibility complex-encoded B-G antigens are found on many cell types that are important for the immune system. Proc. Natl. Acad. Sci. USA **88:** 1359–1363.

Traill K.N., and Wick G. (1983). Chicken thrombocytes. Isolation, serological and functional characterization using the fluorescence activated cell sorter. Dev. and Comp. Immunol. **7:** 111–125.

Weill J.C., and Reynaud C.A. (1987). The chicken B cell compartment. Science **238:** 1094–1098.

